# Association of feed efficiency with organ characteristics and fatty liver haemorrhagic syndrome in laying hens

**DOI:** 10.1038/s41598-023-30007-1

**Published:** 2023-04-11

**Authors:** Doreen Onyinye Anene, Yeasmin Akter, Peter John Groves, Neil Horadagoda, Sonia Yun Liu, Amy Moss, Christine Hutchison, Cormac John O’Shea

**Affiliations:** 1 School of Biosciences, Department of Animal Science, University of Nottingham, Sutton Bonington Campus, Loughborough, LE512RD UK; 2 School of Life and Environmental Sciences (SOLES), Faculty of Science, University of Sydney, Camden, NSW 2570 Australia; 3 Sydney School of Veterinary Science, Faculty of Science, Poultry Research Foundation, University of Sydney, Camden, NSW 2570 Australia; 4 Sydney School of Veterinary Science, Faculty of Science, University Veterinary Teaching Hospital Camden, The University of Sydney, Camden, NSW 2570 Australia; 5 School of Environmental and Rural Science, University of New England, Armidale, NSW 82351 Australia; 6 School of Science, Western Sydney University, Hawkesbury Campus, Richmond, NSW 2753 Australia; 7 Department of Bioveterinary and Microbial Sciences, Technological University of the Shannon: Midlands Midwest-Athlone, Co Westmeath, N37 HD68 Ireland

**Keywords:** Animal physiology, Animal behaviour

## Abstract

Poor feed efficiency (FE) in hens impacts body weight (BW) and may reflect suboptimal health. Fatty Liver Haemorrhagic Syndrome (FLHS) is mostly observed in laying hens and affects egg production and hen performance. The aim of this study was to investigate the relationships of FE and BW with organ characteristics, liver composition and incidence of FLHS of 150 individually housed ISA Brown hens ranked on the basis of feed conversion ratio (FCR) attained from early lay. At 45 weeks, 10 birds per FE group (HFE—High feed efficient; MFE—medium feed efficient; LFE—low feed efficient) were randomly selected and euthanized. Hen BW was positively associated with feed intake and FCR. The HFE hens had a lower abdominal fat pad and liver weight compared to LFE hens. FLHS lesion score was higher (worse) in the LFE than HFE hen group and was moderately positively associated with BW and abdominal fat pad, but strongly positively associated with liver weight. Liver pathology of LFE hens showed hepatocytes with abnormal retention of lipids causing distended cytoplasmic vacuoles compared to the HFE hens. Hens which exhibited poorer FE in early lay had heavier abdominal fat pads, heavier, fatter livers and were more prone to FLHS.

## Introduction

Variation in bodyweight (BW) and poorly controlled feed intake (FI) can lead to poor or inefficient egg production^1,2^. Maintaining BW is a vital objective in hen production systems, as hens weighing above the breed recommendation for their age and phase of production have a poorer feed to egg conversion efficiency^2–6^, come into sexual maturity earlier^7^, produce eggs of lower albumen quality^8^ and have a higher susceptibility to fatty liver haemorrhagic syndrome (FLHS)^9–11^. The liver supports nutrient intake and utilization, lipid and protein synthesis for egg yolk production^12,13^, calcium absorption for eggshell formation^14^ and conversion of toxins into water-soluble waste products excreted by the kidneys^15^. Due to the multiple roles and hence importance of this organ, impaired function may lead to metabolic diseases, reduced productivity, and production of eggs of poor quality.

FLHS is a chronic metabolic disease which has gained attention in recent years as an important health issue in the layer industry. In a survey by Shini et al.^16^, FLHS was reported to cause 74% of the total mortality of caged layers in Queensland, Australia. Harms et al.^17^ and Grimes et al.^18^ showed that hens with FLHS may exhibit with excessively high feed intake, increased body weight, a reduction in egg production and engorged, pale combs. Sudden death also occurs, and at necropsy, the syndrome is characterized by excessive accumulation of fat within the abdominal cavity, a pale and fragile liver sometimes void of structural integrity, multifocal haemorrhages^19^ and large blood clots in the abdominal cavity as a result of liver rupture^20^. Although no diagnostic criteria have been developed to determine FLHS in live birds, it is reported to be induced by the persistence of high oestrogen production^21,22^ and may be seen mostly during the peak production period (between 30 and 40 weeks of age). A certain BW threshold is required to commence follicle development and sexual maturation in hens^23^. At this time, the heightened levels of oestrogen increases the metabolic activity of the liver to form the lipoprotein precursors required for yolk production^13^. Overproduction of lipoproteins by the liver triggers hyperlipidaemia, which is not only a common feature of FLHS, but also causes insulin resistance, lipid peroxidation, and an inflammatory response. This could lead to a loss in liver function and an increased appetite and feed consumption as the hen attempts to maintain egg production, eventually causing obesity, a drop in egg production and a poor feed to egg conversion efficiency.

Recent research suggests that considerable variation in feed intake, BW and feed efficiency exists between individually-housed hens kept under similar management and dietary conditions^24^. These variations have been reported to begin early in the laying period^25^, and are persistent until at least 40 weeks of age^8^. However, there is scarce literature on how these differences in BW and FE established in early lay influence liver health conditions, other organ characteristics, and the incidence of FLHS during peak lay. Therefore, the aim of this study was to explore the associations of FE and BW with organ characteristics and occurrences of FLHS of ISA Brown hens ranked as high, medium, and low feed efficient from early in the laying period under caged conditions.

## Results

### Hen performance

**Figure 1:**
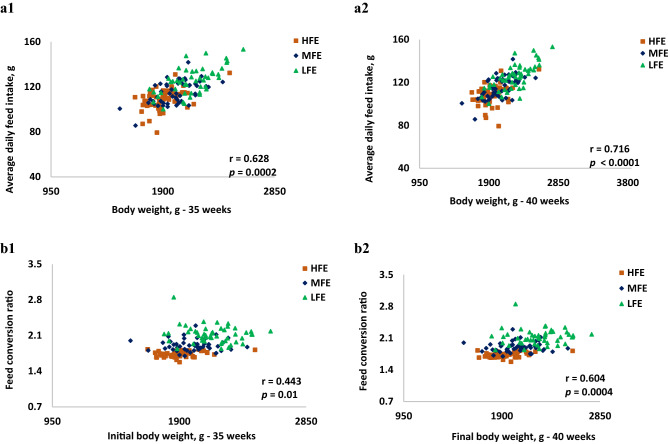
Correlation of average daily feed intake and bodyweights at 35 and 40 weeks old (**a1** and **a2**) and feed conversion ratio and body weights at 35 and 40 weeks old (**b1** and **b2**), of individually housed ISA brown hens. *n* = 150 (50 per FE group). HFE, high feed efficiency; MFE, medium feed efficiency; LFE, low feed efficiency; r = coefficient of correlation, *p* = level of significance.

The performance traits of individually housed hens between 35 and 40 weeks have been reported^8^. Summarily, the hen BW at 35 weeks was higher in the LFE birds compared to the HFE hens (*p* < 0.001) and continued to be heavier than the HFE and MFE hens at 40 weeks. The average daily feed intake was higher and the FCR worst in LFE birds, followed by the MFE and least in the HFE (*p* < 0.001). Egg mass was higher in the HFE (*p* = 0.01), but similar in both MFE and LFE. There were no differences in the egg production and egg weight across the three FE groups. Correlation analysis (*r*) of the hen performance characteristics data from Anene et al.^8^ are presented in Fig. 1. BW at 35 weeks was positively associated with FI (r = 0.628; *p* = 0.0002) and this trend was maintained for BW at 40 weeks (r = 0.716; *p* < 0.0001)*.* The FCR was observed to be positively associated with BW at 35 weeks (r = 0.443; *p* = 0.01) and this was maintained at 40 weeks of age (r = 0.604; *p* = 0.0004) with heavier birds having a higher (worse) FCR compared to lighter weight hens.

### Organ characteristics

The organ characteristics of individually housed ISA Brown hens aged 45 weeks old are presented in Table 1. At 45 weeks, the LFE hens had a higher BW compared to the MFE hens with the lowest BW seen in the HFE hens (*p* < 0.001). The abdominal fat pad weight was different across the three FE groups with LFE hens having the highest fat pad weight and fat pad weight corrected for BW, followed by MFE and the least in the HFE hen group (*p* < 0.001). The LFE and MFE hens had greater liver weights compared to the HFE hens (*p* < 0.001), and this result was consistent when corrected for BW (*p* = 0.04). The total intestinal weight was heavier in the LFE hens followed by MFE hens and least in the HFE hens (*p* < 0.001). However, when corrected for BW, there was no difference across the three FE groups. Although no difference was observed in the absolute weight of the gizzard across the three FE groups in proportion to BW, the gizzard of the HFE hens were seen to be bigger compared to the LFE hens (*p* = 0.01). The absolute pancreas weight, pancreas weight as percentage of BW and pH values of the gizzard, jejunum and ileum were not different across FE groups.

**Table 1:** Organ characteristics of individually housed ISA Brown hens aged 45 weeks old and ranked based of differences in feed efficiency as HFE, MFE and LFE. (n = 30; 10 per FE group).

Organ variables	Feed efficiency groups			SEM	*p-*value
	HFE	MFE	LFE		
BW of bird at 45 weeks of age, g	1852^c^	2190^b^	2371^a^	0.05	< 0.001
Abdominal fat pad weight, g	76^c^	141^b^	184^a^	12.9	< 0.001
Abdominal fat pad weight corrected for BW %	4.1^c^	6.3^b^	7.7^a^	0.43	< 0.001
Liver weight, g	42.9^b^	65.8^a^	67.5^a^	4.91	< 0.001
Liver weight corrected for BW %	2.31^b^	3.01^a^	2.81^a^	0.19	0.04
Intestine weight, g	110^c^	128^b^	144^a^	4.91	< 0.001
Intestine weight corrected for BW %	5.97	5.84	6.12	1.66	0.50
Gizzard weight, g	37.6	40.7	40.8	2.03	0.46
Gizzard weight corrected for BW %	2.03^a^	1.85^ab^	1.71^b^	0.07	0.01
Pancreas weight, g	3.19	3.16	3.70	0.26	0.26
Pancreas weight corrected for BW %	0.17	0.15	0.16	0.01	0.25
Ileal pH	7.90	7.90	7.80	0.16	0.88
Jejunum pH	6.20	6.20	6.50	0.19	0.44
Gizzard pH	4.13	4.10	4.18	0.10	0.86

BW, body weight; HFE, high feed efficiency; MFE, medium feed efficiency; LFE: Low feed efficiency; SEM, standard error of mean; ^abc^—means within rows not sharing a common suffix are significantly different at the 5% level of probability.

**Figure 2:**
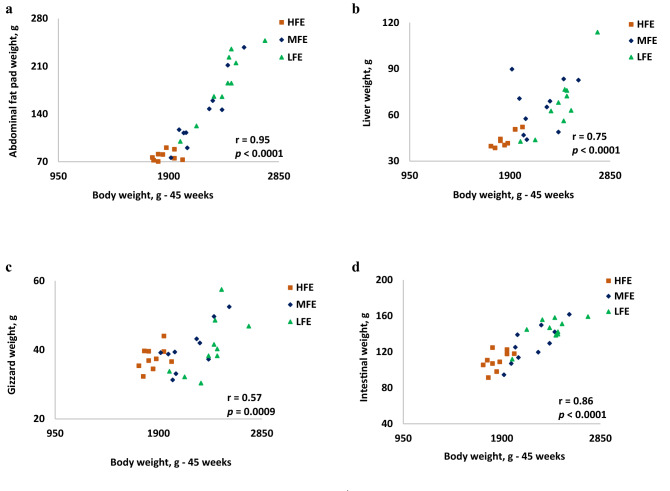
Correlation (*r*) between body weight at 45 weeks and (**a**) abdominal fat pad weight (**b**), liver weight (**c**) gizzard weight and (**d**) total intestinal weight of individually housed hens aged 45 weeks ranked based on differences in feed efficiency. (n = 30; 10 per FE group). HFE, High feed efficiency; MFE, Medium feed efficiency; LFE, Low feed efficiency; r = coefficient of correlation, *p* = level of significance.

Correlation analysis (*r*) between hen performance traits and organ characteristics of individually housed ISA Brown hens (*n* = 30) are presented in Fig. 2 and Table 2. There was a strong and positive association between BW and abdominal fat pad weight, (r = 0.954; *p* < 0.0001), liver weight (r = 0.749; *p* < 0.0001), gizzard weight (r = 0.573; *p* = 0.0009) and intestinal weight (r = 0.857; *p* < 0.0001).

**Table 2:** Correlation (*r*) between performance traits and organ characteristics of individually housed ISA Brown hens aged 45 weeks.

Hen performance traits	Weight (g)					pH		
	Abdominal fat pad	Liver	Intestine	Gizzard	Pancreas	Gizzard	Jejunum	Ileal
FI	0.64***	0.55**	0.76***	0.13	0.25	− 0.06	0.002	− 0.14
EW	− 0.002	0.16	0.13	0.25	− 0.05	0.003	− 0.14	0.17
EM	− 0.15	0.02	0.03	0.23	− 0.19	0.03	− 0.16	0.07
FCR	0.56**	0.34	0.54**	0.05	0.15	0.06	0.35	− 0.25
EP	− 0.26	− 0.19	− 0.12	0.10	− 0.29	0.04	− 0.09	− 0.09

BWC, body weight change; FI, feed intake; EW, egg weight; EM, egg mass; FCR, feed conversion ratio; EP, egg production.

*Coefficients with a superscript of * = *p* ≤ 0.05; ** = *p* < 0.01; *** = *p* < 0.001.

### Hen performance and organ characteristics

Average daily feed intake had a positive correlation with abdominal fat pad weight (r = 0.64, *p* = 0.0001), liver weight (r = 0.55, *p* = 0.002) and total intestinal weight (r = 0.76; *p* < 0.001). FCR was also seen to be positively associated with the abdominal fat pad weight (r = 0.55; *p* = 0.001) and total intestinal weight (r = 0.54; *p* = 0.001). No relationship was observed between production traits and the pH of gizzard, jejunum and ileal of the experimental hens.

### Muscle and liver characteristics

**Figure 3:**
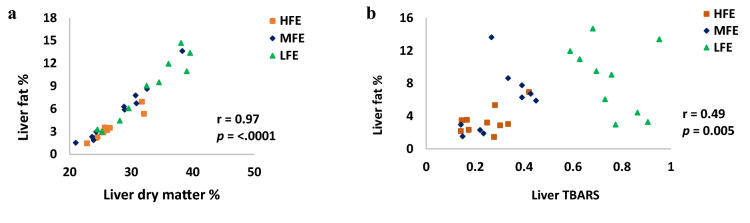
Correlations (*r*) of liver fat with (**a**) liver dry matter and (**b**) liver TBARS value of individually housed hens aged 45 weeks ranked based on differences in feed efficiency (n = 30; 10 per FE group). HFE, high feed efficiency; MFE, medium feed efficiency; LFE, low feed efficiency; r = coefficient of correlation, *p* = level of significance.

As presented in Table 3, the dry matter, total fat composition and colour of the breast muscle (*Pectoralis major*) were not influenced by FE status, however, the livers from the LFE hens were seen to have a higher dry matter percentage (*p* = 0.02) and total fat percentage (*p* = 0.02) compared to the HFE hen group. Liver colour measurements indicated that LFE hens had a higher level of yellowness (b*) value (*p* = 0.004) while lightness (L*) and redness (a*) values were not affected by the differences in FE. As shown in Fig. 3, a moderate positive correlation was seen between total fat content and lipid peroxidation estimate (TBARS) value in the liver (r = 0.49; *p* = 0.005) while a strong positive association was observed between liver dry matter and liver total fats content (r = 0.97; *p* < 0.001), as well as liver b* value and liver fat percentage (r = 0.77; *p* < 0.0001).

**Table 3:** Differences in the muscle and liver composition of individually housed ISA Brown hens aged 45 weeks old, ranked based on feed to egg efficiency (n = 30; 10 hens per group).

Measurements	Feed efficiency			SEM	*p-*value
	HFE	MFE	LFE		
Muscle					
DM%	26.3	26.7	26.4	0.13	0.14
Total fat%	0.26	0.41	0.40	0.07	0.34
Lightness (L*)	54.3	54.2	53.7	0.78	0.85
Redness (a*)	1.67	1.93	2.38	0.33	0.31
Yellowness (b*)	4.57	4.24	4.77	0.38	0.60
Liver					
Dry matter (%)	26.5^b^	28.3^b^	32.7^a^	1.50	0.02
Total fat (%)	3.44^b^	5.77^ab^	8.64^a^	1.08	0.01
Lightness (L*)	41.2	44.4	47.6	1.93	0.08
Redness (a*)	20.7	21.9	22.1	1.06	0.60
Yellowness (b*)	13.9^b^	16.4^b^	20.1^a^	1.18	0.004

HFE, high feed efficiency; MFE, medium feed efficiency; LFE, low feed efficiency; SEM, standard error of mean; ^abc^—means within rows not sharing a common suffix are significantly different at the 5% level of probability.

**Figure 4:**
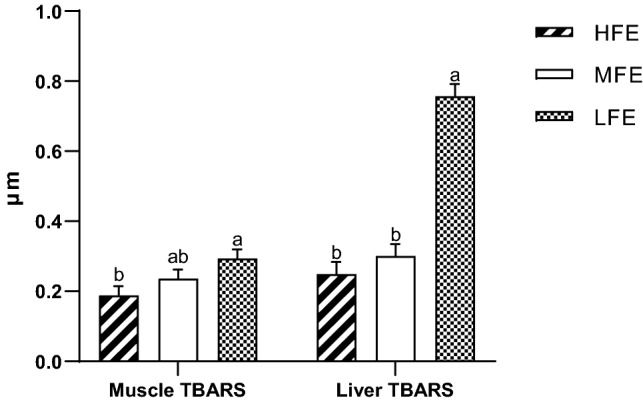
Effect of feed efficiency on the lipid peroxidation estimated of muscle and liver tissues obtained from individually housed ISA Brown hens aged 45 weeks old (n = 30, 10 per FE group). TBARS, thiobarbituric acid reactive substances; HFE, high feed efficiency; MFE, medium feed efficiency; LFE, low feed efficiency; abc Means within rows not sharing a common suffix are significantly different at the 5% level of probability.

The differences in muscle and liver TBARS based on FE status are shown in Fig. 4. Hens in the LFE group had a higher TBARS value in their muscle (*p* = 0.03), and liver (*p* < 0.001) tissues compared to both the MFE and HFE hens.

### Fatty liver haemorrhagic syndrome lesion score and liver histopathology

**Figure 5:**
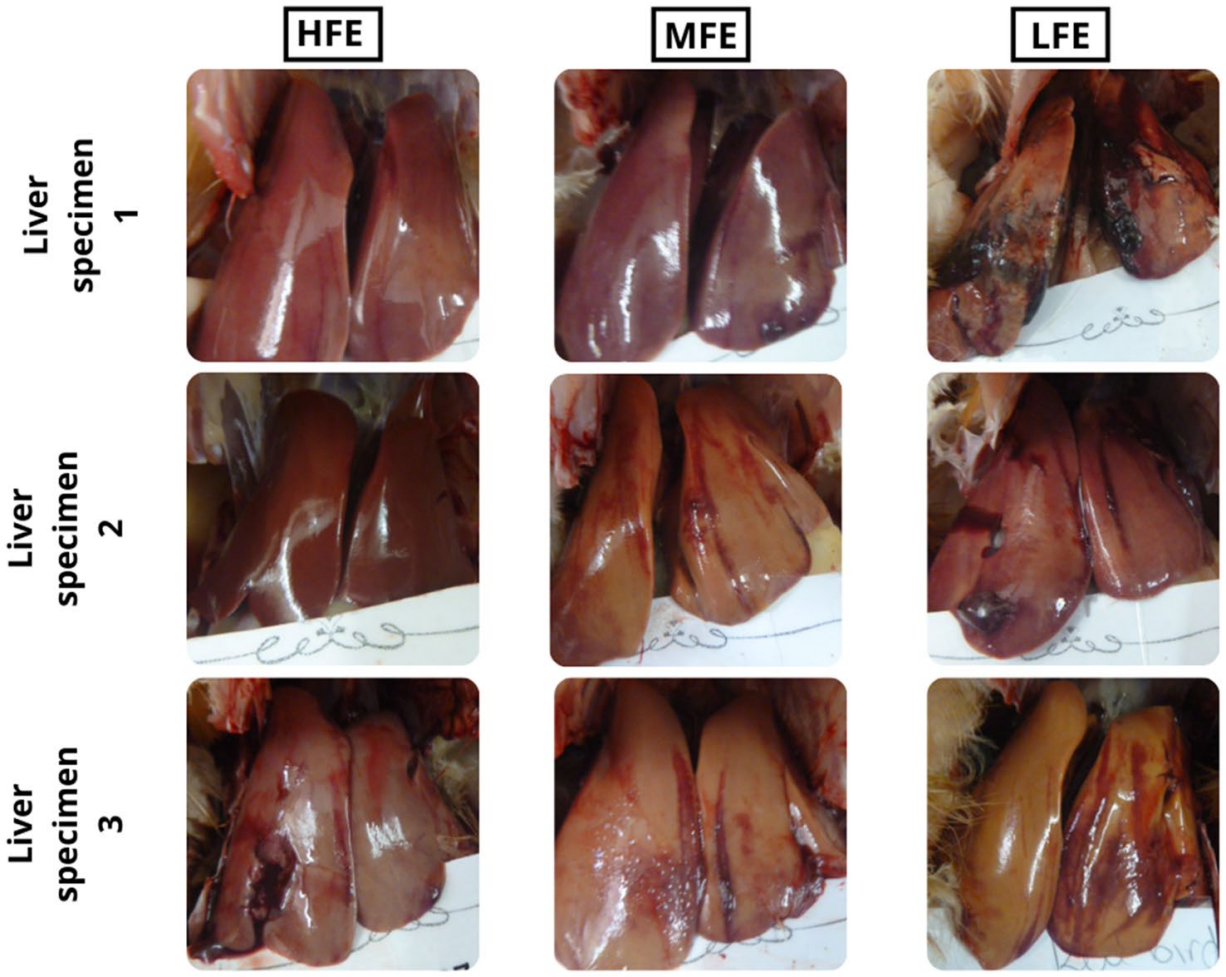
Examples of the physical gross appearance of livers of individually housed ISA Brown hens aged 45 weeks old ranked on the basis of feed efficiency. HFE, high feed efficiency; MFE, medium feed efficiency; LFE, low feed efficiency.

The pictograph of livers obtained from hens of varying FE are presented in Fig. 5. The livers from the LFE hens were seen to be paler and have more haemorrhages compared to the livers from the MFE and HFE hens.

**Figure 6:**
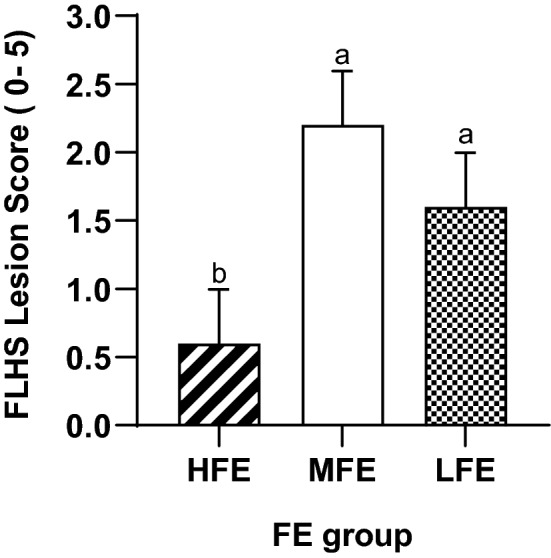
The effect of feed efficiency on FLHS lesions score as seen at necropsy of individually housed ISA Brown hens aged 45 weeks old of varying feed efficiencies (*n* = 30, 10 per FE group). Livers were scored on a scale of 0–5 where higher value indicates greater severity. FE, feed efficiency; FLHS, fatty liver haemorrhagic syndrome; HFE, high feed efficiency; MFE, medium feed efficiency; LFE, low feed efficiency; SEM, standard error of mean; abc Means within rows not sharing a common suffix are significantly different at the 5% level of probability.

The FLHS lesion scoring of individual hens based on FE status are shown in Fig. 6. Hens ranked as HFE had a lower lesion score (0.60 ± 0.40) when compared with both the MFE (2.20 ± 0.40) and LFE group (1.60 ± 0.40).

Correlations (*r*) between some liver characteristics and hen performance variables are presented in Table 4. The results showed that FLHS lesions score had a moderate positive correlation with BW at 45 weeks (r = 0.47, *p* = 0.01). The FI, FCR, abdominal fat pad, liver weight and FLHS lesion score were seen to be positively correlated to liver fat % and liver TBARS. FLHS lesion score had strong positive correlation (r = 0.73, *p* < 0.0001) with liver weight and a moderate association with abdominal fat pad weight (r = 0.48, *p* < 0.01).

**Table 4:** Correlations (*r*) of liver tissue characteristics, organ parameters and performance traits of individually housed ISA Brown hens aged 45 weeks.

Traits	FLHS lesions score	Liver DM %	Liver fats %	Liver TBARS
BW—45 weeks, g	0.47*	0.63***	0.70***	0.60***
FI, g	0.36	0.45*	0.50*	0.62***
FCR	0.34	0.39*	0.42*	0.86***
Liver weight, g	0.73***	0.49*	0.61***	0.26
Abdominal fat pad weight, g	0.48*	0.66***	0.71***	0.63***
FLHS lesions score	-	0.97***	0.77***	0.55**

BW, body weight; FI, feed intake; FLHS, fatty liver haemorrhagic syndrome; FCR, feed conversion ratio; DM, dry matter; TBARS, thiobarbituric acid reactive substances.

*: coefficients with a superscript of * = p ≤ 0.05; ** = *p* < 0.01; *** = *p* < 0.001.

**Figure 7:**
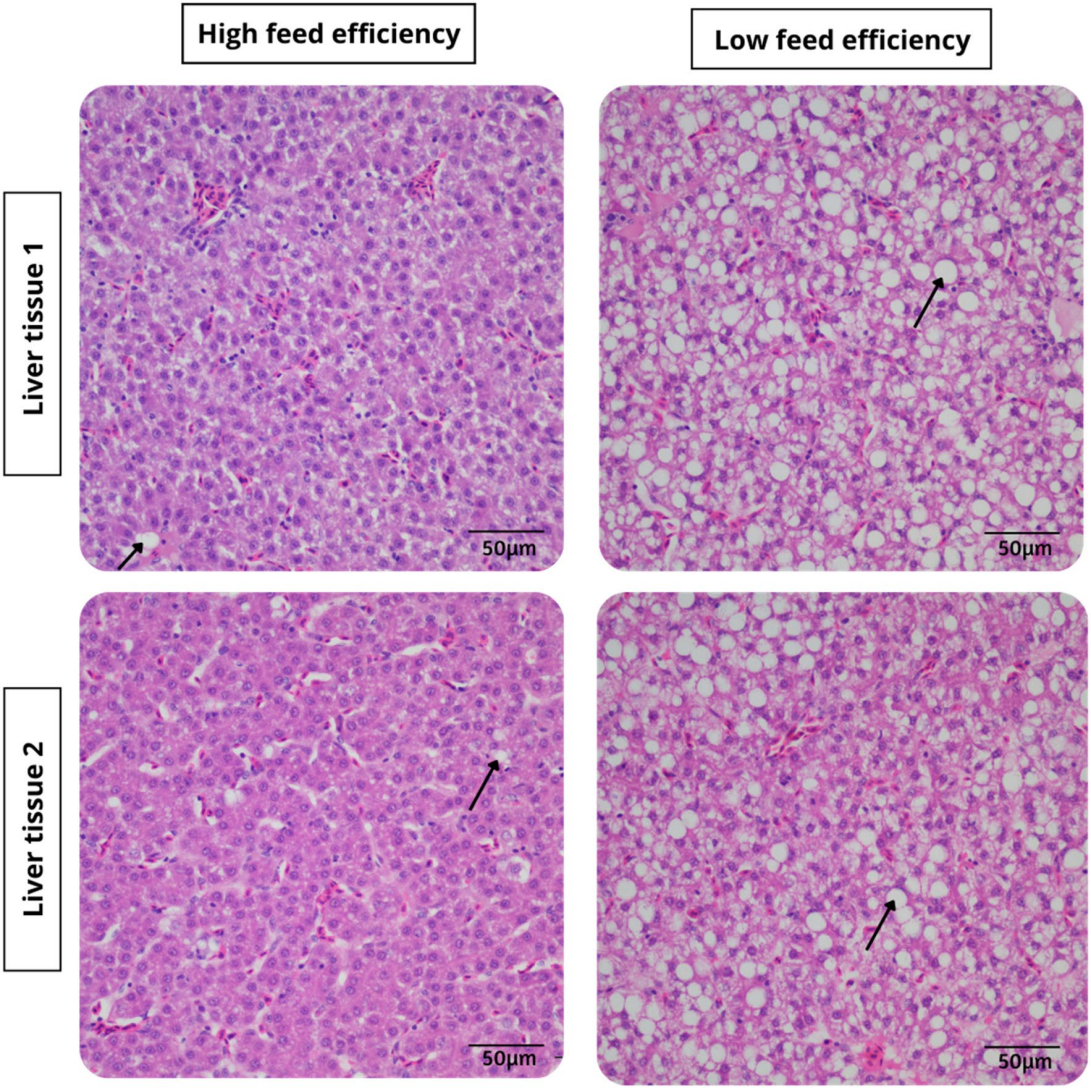
Images of microscopic histological sections of liver tissue after H&E staining, showing liver cells (hepatocytes) of individually housed ISA Brown hens ranked based on feed efficiency. Liver samples were taken when hens were aged 45 weeks. Steatosis within the cytoplasm of the hepatocytes is identified with a black arrow.

The microscopic examination of liver tissue from hens ranked as HFE and LFE are presented in Fig. 7. The HFE liver tissues were seen to contain very little or no cytoplasmic lipid vacuoles compared to the livers from LFE hens that had numerous swollen hepatocytes with distended cytoplasmic lipid vacuoles.

## Discussion

The lack of association between FI and EP agrees with the findings from Harms et al.^26^ and Lacin et al.^2^ which suggests that within the feed intake parameters of this study, feed consumption was not a driver of egg production. Rather, the positive association between feed consumption and BW suggests that in inefficient hens, nutrients and energy which are not used for egg formation will be either excreted or used for BW gain and maintenance. Other authors have reported that changes in BW accounts for about 50–70% of the variation in feed intake^27^. Thus, variation in BW and variation in feed intake are inextricably linked; hens which consume more feed on average tend to gain weight and heavier hens will consume more feed. As shown in this study, hens which are heavier on average and have high feed intake tend to have poor FE rather than produce more or heavier eggs. Thus, flocks whose BW are managed around the breed standard or at a recognized optimal weight for age tend to achieve significant improvements in production performance, with improved persistency of egg production^28^. The heavier abdominal fat pad weight seen in the LFE hens and its positive associations with FI, BW at 45 weeks and FCR confirms that in inefficient hens, energy and nutrients not utilised for egg formation and body maintenance, will be excreted as metabolic waste or heat output or stored as lipids in the abdominal cavity in the form of visceral fat.

The weight of the internal organs and its contribution to the total BW may reflect the health condition of the animal^29^. The higher liver mass seen in the HFE hens of the current study, illustrates that differences in the liver appear to be related to BW and FI. This agrees with previous studies which show that the size of visceral organs are related to the level of FI, as the size of these organs may influence energy requirements for basal metabolism^30^. Interestingly, in this study, the HFE group had a proportionally heavier gizzard, and as a larger gizzard improves nutrient utilisation in chickens, presumably due to a greater functionality^31^., this may explain the better feed efficiency in the HFE hens. The gizzard has also been described as the pace-maker of gut motility which includes episodes of reverse peristalsis that increases the exposure of digesta in the gizzard to proventricular secretions of digestive enzymes^32^. Furthermore, larger, well-functioning gizzards contribute to inhibiting feed overconsumption as it limits the quick passage of feed from the gizzard to the duodenum^33^. Research has shown that satiety can be induced by the stretch and muscular activity of the gizzard^34^ or through humoral signals such as ghrelin, gastrin and cholecystokinin^35^. Another study reported that hyperphagia followed by poor FE within ad libitum fed broiler chickens selected for a high growth rate may only be possible when the gizzard is underdeveloped^36^. Therefore, the higher appetite and subsequent FE seen in the LFE hens of the current study may be linked to the smaller gizzard size. This concept is interesting and the role of targeting gizzard development to drive feed efficiency merits further investigation.

The colour of the liver is an important indicator for poultry viscera health as abnormalities in the colour may be caused by disease conditions^37^. For example, the liver b* value which gives an estimate of yellowness, where a higher value is more yellow, was greatest in LFE birds and corresponded to a higher hepatic fat deposition. According to King and Chen^38^, lipid accumulation in the hepatic cells of the chicken contribute to the intensity of liver colour. Moreover, the studies by Shini and Bryden^19^ and Rozenboim et al.^20^ indicated that a liver with a stronger yellow colour is associated with abundant fat reserves and the hallmarks of FLHS, which include subcapsular and parenchymal haematomas and haemorrhages.

The higher TBARS levels obtained in the LFE hens suggests higher oxidation in the liver tissue due to free radical exposure, which can cause damage to proteins, lipids, and DNA, and can contribute to cell toxicity and lead to the development of FLHS. Oxidative stress, an imbalance between production of free radicals and reactive oxygen species (ROS), leads to damage of important biomolecules and cells, and can potentially impact the whole organism^39^. Liver fat acts as a potential substrate for ROS toxicity and lipid peroxidation^40^. Reactive oxygen species cause lipid peroxidation and cytokine production, contributing to hepatocellular injury and fibrosis^41^, and promote progression from simple steatosis to fatty liver disease^42^. A positive correlation between liver fats and liver TBARS in this study indicates a higher risk of FLHS in the LFE hens due to the presumably greater free radical activity. These results are supported by Rolo et al.^43^ and Ucar et al.^40^, who indicated that lipid peroxidation induces hepatic inflammation which leads to the development of diverse hepatic lesions associated with FLHS. Interestingly, the muscle tissue, which was selected to assess systemic oxidative status, contained elevated levels of TBARS products. This may have implications for general health status and the onset of metabolic conditions.

FLHS is a metabolic disease mostly observed in caged laying hens and associated with high egg production and positive energy balance. The higher FLHS lesion score observed in the LFE hens compared to the HFE hens, suggests that greater feed intake is associated with increased incidences of FLHS. Prolonged consumption of feed in excess of the requirements for egg production and maintenance, such as that observed in the LFE group of the current study, suggests an increased fat accumulation in the liver, which can alter liver function and the FE of laying hens. The FLHS lesion scores in the current study were also associated with greater BW, abdominal fat content, liver weight, liver fat and dry matter content. This is supported by Trott et al.^11^ who reported that approximately 97% of the FLHS affected birds were found to have large fat deposits or were deemed obese. Similarly, King & Chen^38^ reported that a higher chance of FLHS is due to abnormal fat accumulation in the abdominal cavity, the visceral organs and liver cells of the chicken. The study by Whitehead^44^ also reported that increased lipid level of the FLHS birds of up to 47.7% could have a positive effect on dry matter content of the liver. The present study indicates that inefficient hens had greater fat deposition in the liver, thus increasing incidences of metabolic differences in liver function. The increased lipid availability was expressed in the livers of LFE hens by higher TBARS value, as well as swollen hepatocytes noted on microscopic examination of the liver tissue from LFE hens. The excessive accumulation of fat within the hepatocytes weakens the structural integrity of the liver increasing to the risk of haemorrhage. Collectively a picture emerges of FLHS evident mid-lay in high egg producing caged birds and associated with various changes both directly related to the digestive tract and systemically.

## Conclusion

This study shows important relationships between hen BW and FE with organ characteristics and FLHS prevalence in laying hens. Inefficient hens consumed more feed and stored excess energy beyond that required for egg production, which resulted in a positive energy balance leading to greater BW, higher abdominal and liver fat deposition, changes in organ weight and composition and higher incidences of FLHS lesion score compared to HFE hens. Due to the tendency for hens to become overweight when allocated to ad libitum feeding regimes, the management of body weight similar to the recommended breed standards, especially in high producing flocks is critical to the prevention of FLHS. A better understanding of the role of oxidative stress and inflammatory response in the pathogenesis of FLHS would help in the development of new management strategies.

## Materials and methods

This study was conducted at the Poultry Research Foundation, within The University of Sydney, Camden Campus, Australia. All experimental procedures conducted in this study were approved by The University of Sydney Animal Ethics Committee (AEC number, 2017/1212), and were in accordance with the Australian code for the care and use of animals for scientific purposes^45^. The study contained in this manuscript was conducted and is reported in accordance with the ARRIVE guidelines^46^.

### Preluding experimental set up—25 to 30 weeks

The experimental animals, housing conditions, diet composition and performance characteristics measurements used for the selection of hens in this study have been reported in Anene et al.^8^. Briefly, 455 ISA Brown commercial strain pullets were obtained from a certified grower at 16 weeks of age, housed individually in 25 × 50 × 50 cm cages within a layer house, fed a common diet as presented in Supplementary Table S1, and kept under the same environmental conditions. The shed temperature was controlled around 21–24 °C, and photoperiod cycles of 16 h light and 8 h darkness were maintained, as recommended by the ISA Brown management guide. The hens were monitored weekly from 25 to 30 weeks of age for differences in FI, BW, and egg weight, to facilitate subsequent ranking of birds based on FE. All hens were fed a wheat and soybean meal-based mash on an ad libitum basis, which consisted of 16.3% crude protein, 2750 kcal/kg metabolizable energy 0.82% total lysine, 0.42% total methionine, 4.0% calcium and 0.4% available phosphorus to meet the recommendations of the ISA Brown bird from early to mid-lay phase. All hens were offered ad libitum water through water nipples placed in each individual cage. At the end of the 30th week and based on the average feed conversion ratio (FCR) of the preceding six-week period, all 450 hens were ranked into quintiles and the hens in the first, third and fifth quintile were classed into three groups: high feed efficiency (HFE), medium feed efficiency (MFE) and low feed efficiency (LFE). The HFE group had an average FCR of 1.83 ± 0.02, the MFE group had an average FCR of 2.05 ± 0.02 and the LFE group had an average FCR of 2.39 ± 0.02. From 35 to 40 weeks, hen performance characteristics including; FI, egg weight, egg production, egg mass, FCR and change in BW were monitored and calculated as reported in Anene et al.^8^.

### Organ characteristics

At 45 weeks of age, 30 birds from the FE groups (*n* = 10 per FE group) were randomly selected and euthanised by intravenous injection of sodium pentobarbitone via the wing vein. The livers were examined in situ and evidence for FLHS was scored as described by Diaz et al.^47^. Briefly, liver score 0 indicates normal liver, liver score 1 indicates up to 10 subcapsular petechial haemorrhages, liver score 2 indicates more than 10 subcapsular petechial haemorrhages, liver score 3–5 indicates focally extensive to massive subcapsular haemorrhages. The abdominal fat pad, pancreas, gizzard, and whole intestine were excised and weighed individually using an electronic scale with a digital output. Organ weights were also expressed as a percentage of BW. Tissues from the liver and muscle were snap frozen in liquid nitrogen at collection, then stored at − 80 °C until assayed. The pH of digesta within the gizzard was determined in-situ. Digesta from the distal half of the jejunum and ileum were collected in their entirety and pH measured accordingly. The dry matter content of liver and muscle were measured according to AOAC procedures^48^. The total fat content of the liver and muscle were determined by the Ankom XT15 method based on AOCS Official Procedure Am 5-04^49^. Temperature compensated pH values were measured in breast (*Pectoralis major*) muscle at 24 h (ultimate pH) post-mortem as described by Hutchison et al.^50^. Muscle and liver colour were determined using triplicate colour measurements for lightness (L*), redness (a*) and yellowness (b*), (CIELAB, L* a* b* system, illuminant D65, 10° standard observer)^51^. Measurements were taken over the muscle and liver surface at 2.5 cm thick slices using a Minolta Chroma meter (CR-300, Osaka, Japan), after the samples had air-bloomed at 1 °C for 1 h.

### Lipid peroxidation of liver and muscle

Lipid peroxidation of liver and muscle samples were measured as thiobarbituric acid reactive substances (TBARS) using a commercial kit Cayman TBARS (TCA Method) assay kit (Item No. 700870) following the instructions of the manufacturer (Cayman, USA) with minor modifications. Briefly, liver and muscle samples were thawed on ice, chopped into small pieces, and washed twice with ice-cold PBS to remove any blood. A total of 25 mg of tissue samples was transferred in to a 2.0 ml safe lock tube containing two 3 mm diameter metal beads. Then, 250 µl of RIPA buffer with protease inhibitor (EDTA; 10 µl/ml) was added per tube and the sample was homogenized using a Qiagen TissueLyser II lysed at a frequency of 30 Hz for 2 min. The sample was centrifuged at 16,000 × g for 10 min at 4 °C to remove insoluble materials and the supernatants were then collected for measuring TBARS using a Cayman TBARS assay kit.

### Liver histopathology

Liver slices (0.5–1.0 cm) collected at post-mortem examination were fixed in 10% neutral buffered formalin and paraffin wax-embedded 4 µm sections were stained with haematoxylin and eosin (H&E) and assessed microscopically at a magnification of ×40, by a pathologist unaware of the categorisation based on the FCR. Twelve liver sections were reviewed and the degree of hepatocellular vacuolation characterised by clear, well demarcated, single, or multiple cytoplasmic vacuoles was graded as described by Trott et al.^11^. In this grading scheme, sections with no or very rare vacuolation was graded as 0, those with less than 50% of hepatocytes containing vacuoles were graded as 1, liver sections with 50% or greater hepatocyte containing variable size vacuoles or diffuse vacuolation with small vacuoles were graded as 2 while those with diffuse vacuolation with variable sized vacuoles were grade as 3. Multiple liver sections from each bird were assessed to determine the grade. Where there was a difference in the grade between sections for a given case, the predominant change in the sections and evidence of haemorrhage were used for grading.

### Statistical analysis

The individually caged laying hen served as the experimental unit. Experimental data were analysed using the generalised linear model procedure of SAS (SAS Institute) with the FE group as the main effect. All data are presented as least square means ± standard error of the mean (SEM). Differences among least squares means were computed using the pdiff procedure in SAS. Pearson or Spearman correlation coefficients output for production traits and egg quality measurements was generated using the proc corr procedure SAS Institute Inc. The probability value denoting statistical significance was *p* < 0.05.

## Supplementary Information

Supplementary Table S1.

## Data Availability

The data that support the findings of this study are available from the corresponding author upon reasonable request.
