# Supplementary material for: Association of feed efficiency with organ characteristics and fatty liver haemorrhagic syndrome in laying hens
Source: Sci Rep. 2023 Apr 11;13:5872. doi: 10.1038/s41598-023-30007-1 (PMC10090132; doi:10.1038/s41598-023-30007-1)
Supplement: Supplementary file 1 — Supplementary Table S1. [file 41598_2023_30007_MOESM1_ESM.docx]

**Supplementary Table S1: Dietary composition and calculated nutrient composition of the experimental diet (as-fed basis)**

| **Item** | **Amount** |
| --- | --- |
| Feed ingredient, mg/kg |  |
| Wheat (11%) | 312.00 |
| Sorghum (11.5%) | 324.32 |
| Soybean meal (46.5%) | 156.00 |
| Limestone grit (38%) | 70.00 |
| Canola expeller (37%) | 86.00 |
| Limestone | 20.00 |
| Dicalcium phosphate | 15.00 |
| Soybean oil | 9.00 |
| Sodium bicarbonate | 2.40 |
| DL-Methionine | 1.50 |
| Lysine-HCl | 0.50 |
| Salt | 1.8 |
| Layer premix (University of Sydney) ^1^ | 1.00 |
| Choline chloride 60% | 0.30 |
| Ronozyme WX CT | 0.15 |
| Ronozyme Hi-phosphate layer 300 | 0.03 |
| Total | 1,000 |
| Calculated nutrient composition, % |  |
| Crude protein | 16.3 |
| Total digestible lysine | 0.742 |
| Total digestible methionine | 0.397 |
| Total digestible tryptophan | 0.195 |
| Total digestible isoleucine | 0.632 |
| Total digestible arginine | 0.919 |
| Total digestible valine | 0.724 |
| Total digestible threonine | 0.532 |
| Total digestible methionine + cystine | 0.637 |
| Metabolizable energy, kcal/kg | 2750 |
| Crude fat | 2.71 |
| Linoleic acid | 1.40 |
| Calcium | 4.00 |
| Total P | 0.61 |
| Available P | 0.40 |
| Sodium | 0.172 |
| Chloride | 0.174 |
| Crude ash | 13.6 |
| Lysine | 0.838 |
| Methionine | 0.425 |
| Methionine + Cystine | 0.746 |
| Threonine | 0.637 |
| Isoleucine | 0.713 |
| Leucine | 1.505 |
| Tryptophan | 0.225 |
| Arginine | 1.019 |
| Total xanthophyll, mg/kg  Red xanthophyll, mg/kg  Yellow xanthophyll, mg/kg | 6.00  3.10  2.90 |

^1^Each kilogram premix contained vitamin A 15,000 IU; cholecalciferol 1,500 ICU; DL-α-tocopheryl acetate 30 IU; menadione 5.0 mg; thiamine 3.0 mg; riboflavin 6.0 mg; niacin 20.0 mg; pantothenic acid 8.0 mg; pyridoxine 5.0 mg; folic acid 1.0 mg; vitamin B_1_ 15 μg; Mg 80.0 mg; Zn 60.0 mg; Fe 30.0 mg; Cu 5.0 mg; 2.0 mg; and Se 0.15 mg
